# Mutations in EMT-Related Genes in *ALK* Positive Crizotinib Resistant Non-Small Cell Lung Cancers

**DOI:** 10.3390/cancers10010010

**Published:** 2018-01-04

**Authors:** Jiacong Wei, Anthonie J. van der Wekken, Ali Saber, Miente M. Terpstra, Ed Schuuring, Wim Timens, T. Jeroen N. Hiltermann, Harry J. M. Groen, Anke van den Berg, Klaas Kok

**Affiliations:** 1 Department of Genetics, University of Groningen, University Medical Centre Groningen, 9700 RB Groningen, The Netherlands; weijiacong@126.com; 2 Department of Pathology, Shantou University, Shantou 515041, China; 3 Department of Pulmonary Diseases, University of Groningen, University Medical Centre Groningen, 9700 RB Groningen, The Netherlands; a.j.van.der.wekken@umcg.nl (A.J.v.d.W.); t.j.n.hiltermann@umcg.nl (T.J.N.H.); h.j.m.groen@umcg.nl (H.J.M.G.); 4 Department of Pathology and Medical Biology, University of Groningen, University Medical Centre Groningen, 9700 RB Groningen, The Netherlands; a.saber814@gmail.com (A.S.); e.schuuring@umcg.nl (E.S.); w.timens@umcg.nl (W.T.); a.van.den.berg01@umcg.nl (A.v.d.B.); 5 Department of Genetics, University of Groningen, University Medical Centre Groningen, 9700 RB Groningen, The Netherlands; m.m.terpstra.cluster@gmail.com

**Keywords:** whole exome sequencing, adenocarcinoma, *ALK*, crizotinib resistance

## Abstract

Crizotinib is an effective drug for patients with anaplastic lymphoma kinase (ALK)-positive non-small-cell lung cancer (NSCLC), but upon treatment, the tumors inevitably become crizotinib resistant in time. The resistance mechanisms are only partly understood. In this study, we aim to identify gene mutations associated with resistance in ALKpositive advanced non-squamous NSCLC treated with crizotinib. Four ALK positive patients with progressive disease following crizotinib treatment were identified with paired pre- and post-crizotinib tumor tissue from our previously published cohort. Somatic variants in these samples were detected by whole exome sequencing. In one of the four patients, an ALK-resistance associated mutation was identified. In the other three patients, no ALK-resistance associated mutations were present. In these patients we identified 89 relevant somatic mutations in 74 genes that were specific to the resistant tumors. These genes were enriched in 15 pathways. Four pathways, were related to epithelial-mesenchymal transition (EMT): proteoglycans in cancer, HIF-1 signaling, FoxO signaling pathway, and ECM-receptor interaction. Analysis of other EMT-related pathways revealed three additional genes with mutations specific to the crizotinib-resistant tumor samples. The enrichment of mutations in genes associated with EMT-related pathways indicates that loss of epithelial differentiation may represent a relevant resistance mechanism for crizotinib.

## 1. Introduction

Lung cancer is the leading cause of cancer-related deaths worldwide [1]. In the traditional clinical classification, there are two major types of lung cancer, small cell lung cancer, and non-small-cell lung carcinoma (NSCLC) [2]. NSCLC is further subdivided into the major categories of squamous cell carcinoma, adenocarcinoma, and large cell carcinoma, with adenocarcinoma being the most common subtype [2]. Over the last decade, clinical management and treatment of lung cancer patients has become more dependent on molecular classification using “driver” mutations that occur in genes, such as anaplastic lymphoma kinase (*ALK*), epidermal growth factor receptor (*EGFR*), and c-Ros oncogene 1 (*ROS1*) [3,4,5]. Around 5% of all adenocarcinomas have a chromosomal inversion or translocation that produces a fusion product consisting of the kinase domain of ALK combined with EML4 or another fusion partner [5,6]. Tumor cells with an ALK fusion are highly sensitive to tyrosine kinase inhibitors (TKIs) that target ALK, which include crizotinib and the second-generation ALK inhibitors, ceritinib, brigatinib, and alectinib [7,8,9,10]. However, most patients will inevitably acquire resistance to ALK-TKI treatment, usually within one year [11,12].

The mechanisms of resistance can be ALK-dependent or ALK-independent [13]. Secondary *ALK* mutations are observed in 25–33% of patients progressing to crizotinib treatment, and the number of mutations increases to approximately 50% after using a second-generation TKI [14]. Several ALK-independent resistance mechanisms have been proposed based on studies in post-crizotinib tumor samples and cell line models [15,16,17,18,19,20]. These mechanisms include alterations of *EGFR*, proto-oncogene receptor tyrosine kinase *(KIT),* and Insulin like growth factor 1 receptor (*IGF1R)*, as well as activation of the MAP kinase, PI3K/AKT, RAS/MAPK, and JAK/STAT pathways. Studies based on resistant and sensitive cell lines and those based solely on post-resistant tumor tissues have suggested that the epithelial-mesenchymal transition (EMT) potentially mediates resistance against ALK inhibitors [21,22,23]. However, the level of evidence is variable and our understanding of the mechanisms that are involved in crizotinib-resistance remains incomplete.

In this study, we aimed to identify somatic mutations related to crizotinib resistance using whole exome sequencing (WES) of paired tumor biopsies from advanced adenocarcinoma patients, taken both before crizotinib treatment and upon disease progression to crizotinib. 

## 2. Results

### 2.1. Patients

**Figure 1:**
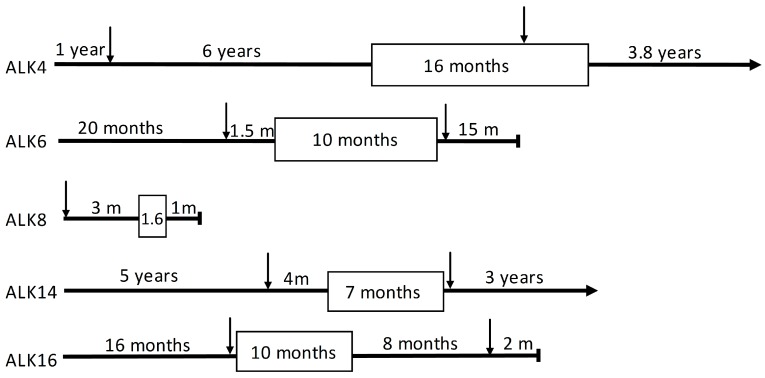
Schematic representation of the total period of treatment and follow-up per patient, indicated by a horizontal black line. ALK4 and ALK14 were alive at the end of study; ALK6, ALK8 and ALK16 died before the end of study). Blocks indicate the duration of crizotinib treatment. Arrows indicate when pre- and post-crizotinib tissue samples were collected. Notes: ALK4 was treated with several lines of chemotherapy and radiotherapy. ALK4’s second biopsy was taken from a local relapse, and crizotinib was continued. Ceritinib was started at further disease progression. Patient ALK6, ALK14, ALK16 received first-line chemo (radio) therapy before the first biopsy. For patient ALK8, crizotinib treatment was stopped after 1.6 months upon disease progression. ALK14 had ongoing response to alectinib subsequent to the crizotinib treatment. ALK16 was treated with ceritinib for eight months following crizotinib treatment.

Four patients (ALK4, ALK6, ALK14, and ALK16) developed crizotinib resistance after initially responding to treatment for approximately one year (Table 1 and Figure 1). One patient, ALK8, did not respond to crizotinib treatment at all and died 2.5 months after initiating crizotinib. ALK6 and ALK16 died 15 months and 10 months, respectively, after crizotinib treatment. At the time of this study, ALK4 and ALK14 were still alive 3.8 and 3 years, respectively, after developing crizotinib resistance. Upon developing resistance, ALK4 was treated with ceritinib and ALK14 with alectinib. Patients ALK6 and ALK16 had not been treated with 2nd or 3rd generation ALK inhibitors, because those drugs were not available at that time.

### 2.2. Whole Exome Sequencing

WES generated an average of 60 × 10^6^ unique reads per sample that passed the Illumina quality filtering steps (Supplementary Table S1). On average, 98% of the unique reads could be aligned to the human reference genome. The mean coverage per sample was 66× with 86% of the target region being covered at least 20×. We identified 582 variants in 519 genes across the four patients who had developed resistance. Of these variants, 20% (116 single-nucleotide variants (SNVs) in 143 genes) had Combined Annotation Dependent Depletion (CADD) scores ≥ 20, 45% (265 SNVs in 253 genes) had CADD scores between 10 and 20, and 26% (151 SNVs in 101 genes) had CADD scores < 10. For 9% of the variants (50 variants in 47 genes), no CADD score was available. Most of the variants without CADD scores (*n* = 46) were small insertions or deletions (INDELs), of which 25 were out of frame INDELs and should be considered as being deleterious. 

We analyzed the validity of the WES data using two independent approaches. First, we reanalyzed RNA-seq data from our published study that included the crizotinib-resistant tumor samples of ALK4 and ALK6 patients [24]. A ≥ 10× coverage at the variant position was observed for 95 out of the 169 WES variants in ALK4 and 34 of the 61 WES variants in ALK6. Ninety out of the 95 variants (95%) in ALK4 and 29 of the 34 variants (85%) in ALK6 were consistent with those that were identified in the WES data, indicating that the majority of the mutant alleles were also expressed in these tumor samples. Our second validation approach was based on an independent WES analysis. After filtering for ≥10× coverage at the variant position, we could evaluate 67 variants in ALK4 and 42 variants in ALK6. We confirmed 61 variants (91%) in ALK4 and 40 variants (95%) in ALK6. When combining both strategies, we independently validated 114 of 123 (93%) variants in ALK4 and 45 of 48 (94%) variants in ALK6.

### 2.3. Variants Related to Crizotinib Treatment

**Figure 2:**
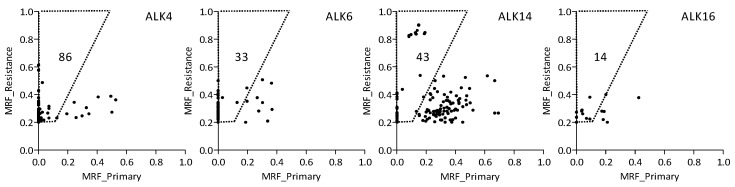
Comparison of mutant read frequencies (MRF) in primary tumor samples (x-axis) and resistant tumor samples (y-axis) from four patients. Each dot represents a single variant. Variants shown in the dashed trapezium have a MRF ≥ 0.20 in resistant samples and at least a two times higher MRF in the treatment-related sample as compared to the primary sample. The total number of “treatment-related” variants in each patient is indicated in the trapezium. In total, 176 variants were identified in 156 genes.

When comparing the primary tumors with the resistant tumors for the four patients with paired samples revealed 175 putatively “treatment-related” variants in 156 genes, of which 136 variants (in 129 genes) were only found in the resistant tumor (Figure 2) (Supplementary Table S2). CADD scores > 20, indicative of deleterious variants, were observed for 16% of these variants (21 SNVs in 15 genes), whereas 43% (75 SNVs in 71 genes) had CADD scores between 10 and 20, 30% (53 SNVs in 49 genes) had CADD scores < 10, and 11% (19 INDELs in 19 genes) had no CADD scores (Supplementary Table S2). Fifteen INDELs caused a frameshift, and can thus be considered deleterious. The distribution of the variants over the different CADD score groups was similar to the distribution observed for all the somatic variants. 

To focus on *ALK*-independent resistance mechanisms, we excluded genes that were mutated in ALK4 because the resistant tumor carried a known *ALK*-resistance-associated mutation (*ALK* G1269A) that explained the crizotinib resistance. The three remaining paired pre- and post-treatment tumors had a total of 90 putatively “treatment-related” variants in 74 genes. Each gene was mutated in only one of the three patients. The 74 genes were involved in 105 pathways. A significant enrichment was observed for 15 pathways in Partek Genomics Suite 6.6 (Supplementary Table S3). Four of these pathways, harboring nine treatment-related mutated genes, were linked to a common biological theme: EMT. These EMT pathways were proteoglycans in cancer, HIF-1 signaling, FoxO signaling, and ECM-receptor interaction (Table 2). Using the Database for Annotation, Visualization and Integrated Discovery (DAVID) pathway analysis tool, we confirmed significant enrichment of proteoglycans in cancer and the HIF-1 signaling pathways, and found borderline significance for the FoxO signaling pathway. Using the Gene Network analysis tool, we found a borderline significant enrichment for the ECM-receptor interaction pathway but also that the other three pathways were not present in Gene Network. Analysis of the other EMT-related pathways in Partek [23] revealed three additional genes with treatment-related mutations (Table 2). In total we found 12 genes uniquely mutated in the resistant tumors that are linked to 13 EMT-related pathways. Variants of six of these genes (*ARNT*, *CTNNA3*, *PTPN11*, *MLTK*, *SMAD4*, and *VEGFA*) had CADD scores higher than 20. Another two genes, *LAMA2* and *MLTK*, had out-of-frame INDELs, which should be damaging. 

In the one non-responding patient, ALK8, we observed somatic variants in three additional EMT-related genes (*ITGAM*, *CACNA1E,* and *RUVBL1*). These three genes are involved in cell adhesion molecules, MAPK signaling, Wnt signaling, and regulation of the actin cytoskeleton. 

Immunohistochemistry (IHC) revealed changes in the E-cadherin and/or Vimentin staining intensity compatible with EMT for only three out of 11 cases (patients ALK20, ALK25, and ALK26). IHC was inconclusive for ALK14 and ALK16. This indicates that other resistant pathways may be operational, although technical issues, such as scarcity of tumor tissue and differences in tissue origin, hampered the reliability of our scoring.

Using a different analysis strategy, performing separate pathway analysis for genes mutated in each individual patient we identified the metabolism pathway as the only pathway that was shared by all five patients for whom we had paired primary and resistant tumor samples. In total, we found 15 variants in 13 genes from this pathway, of which four had CADD scores > 20 and one frameshift insertion. The genes are indicated in Supplementary Table S2. One gene, *CYP11B1*, was also mutated in the non-responding patient ALK8. 

### 2.4. Treatment-Related Copy Number Alterations

We compared WES-based copy number alteration (CNA) plots of the primary tumors to those of their paired resistant samples. Although we did identify several differences in CNAs between primary and resistant samples (Supplementary Figure S1), we did not see a copy number gain in any of the patients for the *ALK*, *MET,* or *KIT* loci known to be associated with resistance. Nor did we observe recurrent CNAs in other parts of the genome shared between these patients.

## 3. Discussion

In this study, we identified 175 variants in 156 genes that are enriched in crizotinib-resistant tumor samples as compared to matched pre-crizotinib samples. In the three patients without *ALK*-resistance-associated mutations, pathway analysis revealed a significant enrichment of nine genes in four EMT pathways. These four pathways were proteoglycans in cancer (*ANK2*, *FASLG*, *HSPG2*, *PTPN11*, *STAT3*, and *VEGFA*), HIF-1 signaling (*ARNT*, *STAT3*, and *VEGFA*), FoxO signaling (*FASLG*, *SMAD4*, and *STAT3*), and ECM-receptor interaction (*HSPG2* and *LAMA2*). No convincing differences in CNAs were observed between the primary and resistant tumor samples, making it unlikely that gene copy number gains were associated with the resistance that was observed in the patients included in this study.

In our study, we have explored ALK-independent mechanisms of crizotinib resistance in advanced NSCLC patients by comparing variants observed in the crizotinib-resistant tumor to the variants present in the tumor before crizotinib treatment. Many mechanisms for crizotinib resistance have been proposed based on cell line studies and studies using resistant samples without comparison to the pre-treatment samples [25,26,27]. These studies revealed activation of several ALK-independent bypass mechanisms, including activation of EGFR, KRAS, SRC, and ERBB and MAPK signaling [25,26,27]. Although these studies generated valuable data, a direct comparison between pre- and post-crizotinib treatment samples is required to truly pinpoint the causal resistance-associated alterations. 

Despite the relatively large number of patients in our previous paper, the total number of patients with matched samples eligible for this study was limited. A general problem in the clinic is the lack of re-biopsies from the growing tumor upon development of resistance. In patients with re-biopsies, the tumor cell percentages are usually low and the biopsy is often fully consumed by the routine diagnostic molecular tests needed to guide further treatment. The limited DNA quality of formalin-fixed paraffin-embedded (FFPE) tumor samples resulted in a suboptimal coverage for part of the exons, which may have prevented the detection of mutations that are present in small subclones of the tumor. However, this should not have affected the identification of treatment-related driver mutations, because they should be present in the majority of the tumor cells in the resistant samples. 

With regard to ALK-dependent mechanisms, we observed an *ALK* G1269A mutation in the resistant tumor of patient ALK4. This mutation was not detected in the primary tumor, as consistent with our previous study using RNAseq on the resistant tumor and ddPCR to analyze the primary tumor sample [14]. This *ALK* gatekeeper mutation has been reported previously in several other studies as a resistance mechanism to crizotinib treatment in *ALK*-positive NSCLC patients [21,28,29].

For the remaining three patients, no *ALK* gatekeeper mutations were found, indicating the activation of *ALK*-independent bypass mechanisms. Here, we focused on treatment-related variants specific for, or with increased mutant read frequencies (MRFs) in, post-treatment samples. In addition to crizotinib treatment, there are several other factors that may have contributed to what we define as treatment-related variants. These factors include treatment history, time interval between biopsies, and differences in anatomic location of the biopsies. Indeed, in patient ALK16, whose biopsies are taken from the same anatomic location, the number of putative treatment-related variants is low. However, development of crizotinib resistance is the common factor in these patients, and thus we can assume that at least a subset of the mutations is directly related to crizotinib resistance. Since there was no commonly mutated gene in these three patients, *ALK*-independent mechanisms appear to be more diverse, a finding that is consistent with the broad variations in resistance mechanisms proposed in the literature [15,28]. To find a possible common mode of action, we performed pathway analysis on the treatment-related resistance genes in these three patients. Four of the significantly enriched pathways were EMT related. Our subsequent analysis of additional EMT-related pathways revealed a total of 12 mutated genes in two of the three patients with ALK-independent resistance mechanisms. Several studies have indicated EMT as a mediator of resistance against ALK inhibitors [21,22,23]. Silencing of Vimentin restored the responsiveness of a crizotinib-resistant cell line to ALK inhibitors [22], whereas in another cell line, the responsiveness to NMS could not be restored [23]. Nevertheless, both of the studies support a role of EMT in crizotinib resistance. Five out of eleven ALK-positive NSCLC patients treated with the second-generation ALK inhibitor ceritinib showed EMT involvement based on immunostaining for E-cadherin and Vimentin [21]. In our study, seven out of the 12 mutated genes involved in EMT had variants that may potentially impact the protein, based on either a CADD score >20 or the presence of out-of-frame INDELs. 

Among the seven mutated genes with high CADD scores, SMAD4 is known to be a crucial regulator of the TGF-beta signaling pathway, which is one of the most important mechanisms leading to EMT [24]. The *SMAD4* W323* mutation observed in ALK6 is in the same functional MH2-domain as the commonly observed R361H and R361C hotspot mutations. This supports a potential causal role for the *SMAD4* W323* mutation. *PTPN11* positively regulates TGF1-induced EMT in lung cancer [30], and the *PTPN11* E76K mutation that is detected in ALK14 was proven to be an activating mutation in lung cancer cells [31]. Overexpression of *VEGFA* in breast cell lines induced SNAIL protein levels, which further reduced E-cadherin expression levels [32]. This suggests that if the *VEGFA* G328R observed in ALK14 is indeed an activating mutation, it may induce loss of E-cadherin. The relevance of the other four genes with high CADD score mutations (*ARNT* F446L, *CTNNA3* E690K, and *MLTK* F157L) or a deleterious out-of-frame deletion (LAMA2 P596Sfs*13) remains unclear. 

Immunostaining for EMT-related protein expression was inconclusive in the subset of available paired pre- and post-treatment samples. This was due to difficulties in scoring caused by differences in tissue origin and scarcity of tumor cells. Moreover, the four EMT-related pathways that are discussed above are also involved in various other biological processes, and these processes might also be related to resistance independent of their association with EMT. Additional cases need to be studied to fully explore the resistance mechanisms that are related to crizotinib resistance. Thus, our data cannot exclude that additional pathways may be involved in the resistance. The metabolism pathway may be a good candidate: it is the only pathway implicated in all five patients and dysregulation of the metabolism pathway has been linked to drug resistance in various types of cancer [33,34]. PDE4D (Ser14Phefs*11), which was mutated in ALK16, was shown to impair cAMP generation through cAMP hydrolysis in lung cancer cell lines. Inhibitors of PDE4 decrease cell proliferation and angiogenesis [35]. Thus, inhibition of the metabolism pathway may provide additional mechanisms for overcoming crizotinib resistance. 

When crizotinib resistance occurs, second-generation ALK inhibitors (ceritinib, alectinib, brigatinib), and, only subsequently, later-generation inhibitors (lorlatinib) are advised as next treatment options, even when the *ALK*-gatekeeper mutation status is unknown [36]. A more optimal approach would be to determine the presence of *ALK*-gatekeeper mutations in progressing tumors in order to justify the choice of the next ALK inhibitor. It is unclear whether tumors that are driven by ALK-independent resistance mechanisms also show tumor response to these drugs. In our study, one of the three patients with an ALK-independent resistance mechanism had a good response to alectinib, suggesting that this drug, in addition to being ALK-specific, may target ALK-independent resistance mechanisms [37,38].

## 4. Materials and Methods

### 4.1. Patient Inclusion

In a previous study we described 29 *ALK*-positive non-squamous NSCLC patients who were treated with crizotinib [12]. Their *ALK* status was assessed by immunohistochemistry. Patients involved in our earlier study gave informed consent for the use of their biopsies for further analysis. For four of these 29 patients, we had sufficient FFPE or frozen pre- and post-treatment tumor material to carry out WES. The four patients are designated ALK4, ALK6, ALK14, and ALK16 in concordance with our previous study [12]. In addition to these samples, we also had FFPE tumor material from one patient, ALK8, who had no response to crizotinib. We had previously found an *ALK* G1269A gatekeeper mutation in ALK4 by RNA-sequencing of the resistant tumor sample of this patient [14]. For each patient, white blood cells were isolated from peripheral venous blood using standard protocols. Tumor samples from patient ALK14 before crizotinib treatment and from patients ALK4 and ALK6 upon developing resistance to crizotinib treatment were fresh frozen. All the other tumor specimens were FFPE. Macrodissection was applied to the tumor samples to achieve tumor cell content greater than 60%. Patients provided informed consent for procurement of extra tumor tissue and analysis of tissue specimen and was approved by the Medical Ethical Committee.

### 4.2. DNA Isolation

DNA was isolated from blood and frozen samples using a standard salt-chloroform DNA isolation protocol. For FFPE samples, DNA was isolated using the ReliaPrep™ FFPE gDNA Miniprep System kit (Promega, Madison, WI, USA) following the manufacturer’s protocol. DNA concentrations were measured by NanoDrop (Thermo Fisher Scientific Inc., Waltham, MA, USA), and DNA quality was evaluated on a 1% agarose gel. 

### 4.3. Whole Exome Sequencing

WES was carried out on 0.6–2 μg genomic DNA of normal and tumor-derived DNA samples (BGI Tech Solutions Co. Ltd., Hong Kong, China). Target enrichment was done using the Agilent SureSelect All Exon V5 kit (Agilent technologies, Santa Clara, CA, USA). Paired-end sequencing with a read length of 2 × 90 nt was performed on Illumina HiSeq2000. 

As a part of the validation procedure, we performed WES a second time on the crizotinib-resistant samples from patients ALK4 and ALK6, following our previously published protocol and data analysis pipeline [14]. RNA-sequencing data from ALK4 and ALK6 resistant samples were re-analyzed to confirm the presence of mutations [14]. 

### 4.4. Immunohistochemistry

Eleven paired formalin-fixed tissue samples from our previous study (ALK1, ALK4, ALK5, ALK6, ALK14, ALK16, ALK17, ALK20, ALK22, ALK25, and ALK26) were used for IHC staining of two EMT biomarkers: E-cadherin (using mouse monoclonal E-cadherin 1:100, clone 36/E-cadherin; BD Biosciences, Breda, The Netherlands) and Vimentin (using mouse monoclonal Vimentin 1:100, clone sc-6260; Santa Cruz Biotechnology; Bioconnect, Huissen, The Netherlands). 

### 4.5. Data Analysis

Raw reads were processed using our in-house pipeline, as described previously (24). In brief, our variant calling pipeline is based on the Genome Analysis Toolkit (GATK) workflow and uses Molgenis Compute as workflow management software [39]. Read alignment was done using Burrows-Wheeler Aligner and GATK, using the human genome reference build GRCh37 with decoys from the GATK bundle [39,40]. Picard Tools was used for format conversion and marking duplicates. HaplotypeCaller was used for integrated calling of the variants for all of the samples from the same patient/cohort. Variants were annotated using SnpEff/SnpSift with the Ensembl release 75 gene annotations and the dbNSFP2.7 database [41]. GATK was used to identify variants. Variants were annotated with dbsnp 138, Cosmic v72, 1000 genomes phase 3 and the ExAC 0.3 databases [42,43,44]. The data were filtered for quality metrics similarly to GATK recommendations using custom filters for population frequency and variant effect. Based on the presence of variants in the normal sample, germline variants were excluded from further analysis. Additionally variants were excluded if present in 100 genomes phase 3 with a minor allele frequency > 0.02.

CADD scores were used to predict pathogenicity of the identified variants. Variants with a CADD score ≥ 20 are defined as deleterious, those between 10 and 20 possibly deleterious, and those <10 non-deleterious [45]. 

To identify somatic mutations we first excluded variants for which the total number of reads in the normal sample was < 10. Next, we excluded all of the variants for which one or more mutant reads were present in the normal sample. We then excluded variants with total reads < 10 in either the primary or the resistant samples. The remaining variants, with two or more mutant reads in either the pre-treatment tumor or post-treatment tumor samples, were considered somatic. Variants detected in the resistant samples with MRFs ≥ 0.20, and for which the MRF is at least twice the MRF in the paired primary sample, are indicated as “treatment-related” variants. 

Pathways overrepresented in the set of genes mutated specifically in the crizotinib-resistance tumor samples were identified with the Partek Genomics Suite 6.6 software package (Partek Inc., St. Louis, MO, USA). In addition, we used Gene Network [46] and DAVID v6.8 [47] to validate our findings by Partek.

### 4.6. WES-Based Analysis of CNAs

Pseudo probe data were generated with SAMtools VarScan2 and DNAcopy [48,49,50]. Briefly, when frozen tumor tissue was used for the isolation of high quality DNA samples, pseudo-probe-derived GC-normalized log2 copy number ratios were generated for each sample using the corresponding normal sample. For FFPE tumor samples, we used WES data of a merged pool of normal FFPE samples as the reference. All of the alignments with a mapping quality > 40, in combination with a minimal segment size of 2 kb and a maximal segment size of 5 kb, with a mean base-wise coverage of at least 1×, were used to calculate the ratios. DNA copy number variant calls were compared between the resistant and the primary tumor sample based on assigned ploidity and by visual inspection of the copy number changes.

## 5. Conclusions

In conclusion, we have identified mutations in genes involved in EMT-related and metabolism pathways in crizotinib-resistant tumors without *ALK*-gatekeeper mutations. From a clinical perspective, the mutational status of patients may provide therapeutic guidance for clinical management of their NSCLC by targeting loss of epithelial differentiation and/or metabolism pathways. 

## Figures and Tables

**Table 1:** Characteristics of the five anaplastic lymphoma kinase (ALK) positive advanced non-small-cell lung cancer (NSCLC) patients.

Patient	Gender	Age at Diagnosis (Years)	PFS ^1^ (m)	Smoking Status (py ^2^)	Tumor Location	
					Primary ^4^	Resistant ^5^
ALK4	female	34	15.9	non-smoker	ovary	liver
ALK6	male	55	9.5	past-smoker (15)	cervical lymph node	glenoid
ALK8	male	76	1.6	past-smoker (NA ^3^)	lymph node mediastinal 4 right	not done
ALK14	female	62	8.4	current smoker (20)	brain occipital metastasis	mediastinal lymph node 7
ALK16	female	48	5.1	past-smoker (18)	lung	lung

^1^ PFS: progression-free survival (time from start of treatment to disease progression estimated by CT); ^2^ py: pack years; ^3^ NA: not available; ^4^ Primary: primary tumor biopsy; ^5^ Resistant: resistant tumor biopsy.

**Table 2:** Overview of all epithelial-mesenchymal transition (EMT)-related pathways that include genes with resistance-related mutated in patients ALK6, ALK14, and ALK16.

Pathway Name	Patient(s)	Genes Mutated	Enrichment Score	Enrichment *p*-Value ^1^
Proteoglycans in cancer ^2^	ALK6 ALK14	*ANK2*, *FASLG*, *HSPG2*, *PTPN11*, *STAT3*, *VEGFA*	8.0	0.00
HIF-1 signaling pathway ^2^	ALK6 ALK14	*ARNT*, *STAT3*, *VEGFA*	4.3	0.01
FoxO signaling pathway ^2^	ALK6 ALK14	*FASLG*, *SMAD4*, *STAT3*	3.7	0.03
ECM-receptor interaction ^3^	ALK6 ALK14	*HSPG2*, *LAMA2*	3.3	0.04
Adherens junction ^3^	ALK6 ALK14	***CTNNA3***, *SMAD4*	3.0	0.05
Ras signaling pathway	ALK14	*FASLG*, *PTPN11*, *VEGFA*	2.7	0.07
Jak-STAT signaling pathway	ALK6 ALK14	*PTPN11*, *STAT3*	1.9	ns
PI3K-AKT signaling pathway	ALK14	*FASLG*, *LAMA2*, *VEGFA*	1.8	ns
Focal adhesion ^3^	ALK14	*LAMA2*, *VEGFA*	1.6	ns
VEGF signaling pathway ^3^	ALK14	*VEGFA*	1.3	ns
TGF-beta signaling pathway	ALK6	*SMAD4*	1.2	ns
MAPK signaling pathway	ALK14	*FASLG*, ***MAP3K20***	1.1	ns
Wnt signaling pathway ^3^	ALK6	*SMAD4*	1.0	ns
Regulation of actin cytoskeleton	ALK6	***SCIN***	0.5	ns

^1^ *p*-value based on analysis with Partek; ns, not significant; ^2^ Borderline or significant in DAVID; ^3^ Borderline or significant in Gene Network; Bold type indicates additional genes not included in the four pathways significantly enriched in Partek.

## Supplementary Materials

The following are available online at http://www.mdpi.com/2072-6694/10/1/10/s1. Figure S1: Copy number variant plots across four patients with *ALK* rearranged advanced NSCLC with matched primary and resistant tumor samples. Table S1: Whole exome sequencing data in five patient samples; Table S2: Treatment-related variants in all tumor samples in ALK4, ALK6, ALK14, and ALK16; Table S3: Significantly enriched pathways harboring treatment-related variants in patients ALK6, ALK14 and ALK16.
