# Supplementary material for: Mutations in EMT-Related Genes in ALK Positive Crizotinib Resistant Non-Small Cell Lung Cancers
Source: Cancers (Basel). 2018 Jan 4;10(1):10. doi: 10.3390/cancers10010010 (PMC5789360; doi:10.3390/cancers10010010)
Supplement: Supplementary file 1 [file cancers-10-00010-s001.pdf]

Mutations in EMT-Related Genes in *ALK* Positive Crizotinib Resistant Non-Small Cell Lung Cancers

Jiacong Wei, Anthonie J. van der Wekken, Ali Saber, Miente M. Terpstra, Ed Schuuring, Wim Timens, T. Jeroen N. Hiltermann, Harry J. M. Groen, Anke van den Berg and Klaas Kok

Supplementary Table S1. Whole exome sequencing data in five patient samples.

Sample		ALK4			ALK6			ALK8		ALK14			ALK16		
		N	P	R	N	P	R	N	R	N	P	R	N	P	R
Clean reads x10^6		61	74	64	60	66	65	58	59	59	58	57	61	142	61
Unique reads x10^6		58	62	60	57	59	60	57	58	56	56	55	58	82	60
Percentage of unique reads		95%	84%	94%	95%	89%	92%	97%	97%	96%	97%	96%	96%	58%	98%
Unique aligned reads (x10^6)		57	60	59	56	58	58	56	56	55	55	54	57	79	59
Percentage of unique aligned reads		98%	97%	98%	98%	98%	98%	98%	98%	98%	98%	98%	98%	96%	98%
Average target coverage		68	70	66	65	71	68	65	65	68	65	65	65	63	66
20X coverage		91%	90%	91%	91%	90%	91%	90%	65%	91%	89%	74%	91%	91%	73%
Number of variants		0	82	169	0	44	61	0	31	0	168	180	0	29	38
CADD Scores	<10	0	20	28	0	13	15	0	5	0	34	45	0	4	6
	[10,20)	0	34	89	0	17	23	0	16	0	77	77	0	14	14
	≥20	0	18	46	0	8	13	0	6	0	50	46	0	7	9
	NA	0	10	6	0	6	10	0	4	0	7	12	0	4	9

N: Normal sample; P: Primary tumour sample before the start of treatment with crizotinib; R: Resistant tumour sample taken when disease progression was observed by CT or PET/CT during crizotinib treatment.

Supplementary Table S2. Treatment-related variants in all tumor samples in ALK4, ALK6, ALK14, and ALK16.

Chr	Position (HG19)	REF	ALT	Gene symbol	Variant Type	Amino acid change[1]	CADD	Primary sample		Resistant sample		Involvement in EMT or metabolism pathways
								Altered reads	Total reads	Altered reads	Total reads	
**ALK4**												
1	1254808	G	C	*CPSF3L*	SNV	p.(Ile105Met)	20	0	99	9	44	
1	2435753	C	G	*PLCH2*	SNV	p.(Pro1118Ala)	1	0	76	23	94	Metabolism
1	110921947	C	A	*SLC16A4*	SNV	p.(Leu186Phe)	16	0	31	14	41	
1	149858085	C	T	*HIST2H2BE*	SNV	p.(Glu36Lys)	35	0	132	21	105	
1	161736226	G	C	*ATF6*	SNV	p.(Asp26His)	19	0	100	14	52	
1	173703378	G	C	*KLHL20*	SNV	p.(Glu184Gln)	29	0	137	43	178	
1	175048588	G	A	*TNN*	SNV	p.(Gly177Ser)	11	0	134	15	73	EMT[2]
2	28762018	C	T	*PLB1*	SNV	p.(Ser224Phe)	15	1	39	19	39	Metabolism
2	28841228	G	C	*PLB1*	SNV	p.(Asp1093His)	19	0	53	11	39	Metabolism
2	97377492	C	T	*LMAN2L*	SNV	p.(Asp240Asn)	31	0	73	20	60	
2	102413796	C	CTTT G	*MAP4K4*	Frameshift Insertion	p.(His26Phefs*3)	NA	0	33	8	35	EMT
2	202208931	C	G	*ALS2CR12*	SNV	p.(Glu142Gln)	19	0	79	26	90	
2	202939778	G	A	*AC079354.1*	SNV	p.(Trp83*)	34	0	25	11	39	
2	211447349	G	T	*CPS1*	SNV	p.(Met185Ile)	10	0	131	32	111	Metabolism
2	225360551	C	T	*CUL3*	SNV	p.(Glu614Lys)	15	2	76	18	66	
2	234978605	G	A	*SPP2*	SNV	p.(Arg195Lys)	18	0	52	16	65	
3	15084402	G	C	*NR2C2*	SNV	p.(Glu579Gln)	30	0	78	18	70	
3	48541457	G	A	*SHISA5*	SNV	p.(Pro24Leu)	18	0	79	7	35	
3	50290516	G	A	*GNAI2*	SNV	p.(Asp122Asn)	23	0	151	16	79	
3	52397143	G	A	*DNAH1*	SNV	p.(Glu1743Lys)	34	0	17	11	41	
3	133486927	G	T	*TF*	SNV	p.(Cys514Phe)	17	0	39	10	45	EMT
3	182988049	G	C	*B3GNT5*	SNV	p.(Asp155His)	16	0	80	23	97	Metabolism
3	190127781	C	T	*CLDN16*	SNV	p.(Arg292Cys)	15	0	53	15	43	EMT
6	31655514	G	C	*ABHD16A*	SNV	p.(Ser484*)	38	0	81	23	104	
6	109768619	C	T	*MICAL1*	SNV	p.(Glu690Lys)	10	0	42	21	60	
7	4153039	G	A	*SDK1*	SNV	p.(Ala1185Thr)	26	2	206	34	115	
7	99768020	G	A	*GPC2*	SNV	p.(Arg525Trp)	17	0	133	14	68	
8	11606590	G	A	*GATA4*	SNV	p.(Arg261Gln)	31	2	107	12	56	EMT
8	23709762	T	C	*STC1*	SNV	p.(Asp85Gly)	23	0	52	10	49	
8	38034637	C	T	*BAG4*	SNV	p.(Arg84*)	37	1	141	21	91	

9	33385784	C	G	*AQP7*	SNV	p.(Glu202Asp)	11	1	14	6	19	
9	33385786	C	G	*AQP7*	SNV	p.(Glu202Gln)	4	1	14	6	20	
9	75773645	C	T	*ANXA1*	SNV	p.(Ser34Leu)	12	1	57	13	56	
9	119249702	C	G	*ASTN2*	SNV	p.(Glu1145Gln)	11	0	56	12	34	
10	20357108	C	G	*PLXDC2*	SNV	p.(Leu161Val)	27	1	53	11	51	
10	65358936	G	C	*REEP3*	SNV	p.(Asp104His)	20	0	128	26	114	
10	94373137	G	A	*KIF11*	SNV	p.(Asp265Asn)	34	0	48	18	52	
10	114925655	C	G	*TCF7L2*	SNV	p.(Ser595Cys)	16	0	250	35	163	EMT
11	3060438	G	A	*CARS*	SNV	p.(Gln213*)	14	0	79	13	48	
11	6416928	G	A	*APBB1*	SNV	p.(Arg657Cys)	19	0	101	20	85	
11	47602214	G	A	*NDUFS3*	SNV	p.(Glu91Lys)	15	1	81	31	117	Metabolism
11	57191474	G	C	*SLC43A3*	SNV	p.(Ile127Met)	1	0	50	12	41	
11	72410500	C	G	*ARAP1*	SNV	p.(Glu800Asp)	12	0	50	18	61	
11	117117615	G	T	*RNF214*	SNV	p.(Glu304*)	34	0	121	73	119	
11	124857614	C	G	*CCDC15*	SNV	p.(Gln498Glu)	11	0	119	64	250	
11	129762618	G	C	*NFRKB*	SNV	p.(Leu56Val)	13	0	13	5	21	
12	10213832	G	A	*CLEC9A*	SNV	p.(Met93Ile)	4	0	40	17	60	
12	27849943	G	C	*REP15*	SNV	p.(Glu150Gln)	19	0	57	33	95	
12	43763100	C	T	*ADAMTS20*	SNV	p.(Cys1844Tyr)	17	0	32	6	22	
12	53445723	G	A	*TENC1*	SNV	p.(Asp64Asn)	33	0	49	34	90	
12	110206933	C	T	*FAM222A*	SNV	p.(Ser400Leu)	17	0	89	49	85	
14	21424243	G	C	*RNASE2*	SNV	p.(Val105Leu)	15	0	74	28	103	
14	64694303	G	A	*ESR2*	SNV	p.(Ser479Leu)	11	0	107	34	130	
14	102551674	C	G	*HSP90AA1*	SNV	p.(Lys330Asn)	17	0	14	3	15	EMT
15	51829933	C	A	*DMXL2*	SNV	p.(Glu457*)	38	0	92	16	54	
15	52404866	C	T	*BCL2L10*	SNV	p.(Glu20Lys)	12	0	67	10	44	
15	78927783	C	T	*CHRNB4*	SNV	p.(Val68Met)	19	1	28	5	23	
15	85360163	G	A	*ALPK3*	SNV	p.(Arg29Lys)	12	1	103	14	67	
16	14758921	G	A	*BFAR*	SNV	p.(Glu385Lys)	28	0	16	6	28	
16	67858669	C	T	*TSNAXIP1*	SNV	p.(Ser222Leu)	29	0	41	21	87	
16	68894173	C	G	*TANGO6*	SNV	p.(Leu161Val)	18	0	81	26	80	
17	6014162	G	A	*WSCD1*	SNV	p.(Gly361Arg)	26	0	31	12	52	
17	6014163	G	A	*WSCD1*	SNV	p.(Gly361Glu)	25	0	33	12	52	
17	7577106	G	T	*TP53*	SNV	p.(Pro278Thr)	29	0	65	11	50	Metabolism; EMT
17	9515661	C	T	*WDR16*	SNV	p.(Thr307Ile)	17	0	42	11	55	
17	28706662	G	A	*CPD*	SNV	p.(Asp222Asn)	26	0	140	12	57	
17	37596699	C	G	*MED1*	SNV	p.(Gln113His)	11	0	66	17	66	
17	56389316	C	T	*BZRAP1*	SNV	p.(Glu956Lys)	28	0	16	8	27	

17	56833643	TGAG	T	*PPM1E*	Inframe Deletion	p.(Glu100del)	NA	1	14	3	13	
17	61623172	C	A	*KCNH6*	SNV	p.(Ser965Tyr)	22	0	74	26	123	
17	66042632	C	G	*KPNA2*	SNV	p.(Gln504Glu)	2	0	59	10	46	
17	77769081	C	T	*CBX8*	SNV	p.(Glu175Lys)	13	0	131	17	76	
18	3499195	C	A	*DLGAP1*	SNV	p.(Gln974His)	21	0	13	6	14	
18	3499203	C	A	*DLGAP1*	SNV	p.(Glu972*)	45	0	18	6	18	
18	60225942	G	C	*ZCCHC2*	SNV	p.(Lys477Asn)	19	0	14	4	16	
18	60999067	C	T	*KDSR*	SNV	p.(Arg316His)	36	0	43	29	84	Metabolism
19	1993141	C	G	*BTBD2*	SNV	p.(Glu188Gln)	28	0	200	24	95	
19	2252675	C	T	*JSRP1*	SNV	p.(Glu217Lys)	14	0	133	23	108	
19	6375401	C	A	*PSPN*	SNV	p.(Ala154Ser)	8	1	16	4	15	
19	17881285	G	A	*FCHO1*	SNV	p.(Val137Ile)	12	0	54	10	39	
19	50762440	C	T	*MYH14*	SNV	p.(Arg758Cys)	17	0	101	15	57	EMT
19	55697677	C	G	*PTPRH*	SNV	p.(Gln898His)	7	0	71	17	82	
19	55823305	C	T	*BRSK1*	SNV	p.(Pro752Ser)	14	0	83	7	33	
20	34091835	C	T	*CEP250*	SNV	p.(Arg1880Trp)	17	0	68	19	56	
20	54961418	G	C	*AURKA*	SNV	p.(Gln72Glu)	1	0	18	8	36	
22	32875149	G	A	*FBXO7*	SNV	p.(Glu102Lys)	20	0	75	35	146	
**ALK6**												
1	22161357	G	A	*HSPG2*	SNV	p.(Ala3512Val)	15	0	143	49	159	EMT
1	32221795	C	A	*BAI2*	SNV	p.(Ala215Ser)	10	1	33	22	58	
1	33960601	AG	A	*ZSCAN20*	Frameshift Deletion	p.(Ser887Alafs*176)	NA	0	62	25	79	
1	108686276	A	C	*SLC25A24*	SNV	p.(Asp329Glu)	19	0	94	35	133	
1	145457071	C	T	*POLR3GL*	SNV	p.(Glu164Lys)	19	0	26	13	26	Metabolism
2	99787087	C	A	*MITD1*	SNV	p.(Gly169Val)	6	0	144	26	93	
2	242681917	G	A	*D2HGDH*	SNV	p.(Val140Ile)	24	0	105	27	89	
3	11059618	A	AT	*SLC6A1*	Frameshift Insertion	p.(Gly111Argfs*96)	NA	0	26	9	41	
3	45943064	G	A	*CCR9*	SNV	p.(Val262Ile)	19	0	117	54	172	
3	56041309	G	C	*ERC2*	SNV	p.(Ala654Gly)	23	0	59	10	41	
4	82366740	TGAAATA	T	*RASGEF1B*	Inframe Deletion	p.(Tyr288_Phe289del)	NA	0	114	24	105	
4	114279156	A	T	*ANK2*	SNV	p.(Ser3128Cys)	16	0	71	21	85	EMT
5	33947471	T	A	*SLC45A2*	SNV	p.(Lys389*)	32	9	46	26	58	
5	140798719	G	GT	*PCDHGB7*	Frameshift Insertion	p.(Leu432Phefs*13)	NA	0	232	56	167	

5	178310282	A	G	*ZNF354B*	SNV	p.(Lys277Glu)	17	0	81	32	93	
7	12620815	A	G	*SCIN*	SNV	p.(Lys162Arg)	14	0	35	9	21	EMT
7	12620816	G	T	*SCIN*	SNV	p.(Lys162Asn)	11	0	36	9	22	EMT
8	131124478	C	A	*ASAP1*	SNV	p.(Gly755*)	44	0	175	30	110	
11	5632354	G	T	*TRIM6*	SNV	p.(Asp445Tyr)	15	0	79	28	92	
12	27915919	G	GT	*MANSC4*	Frameshift Insertion	p.(Leu259Thrfs*9)	NA	0	76	38	141	
12	55846835	G	A	*OR6C2*	SNV	p.(Ala280Thr)	16	0	103	35	124	
12	88383137	AAC	A	*C12orf50*	Frameshift Deletion	p.(Cys201Leufs*13)	NA	0	106	38	136	
12	108986065	G	A	*TMEM119*	SNV	p.(Ala32Val)	12	0	12	4	17	
12	125292384	C	T	*SCARB1*	SNV	p.(Gly311Glu)	25	0	66	16	62	
14	31619345	C	A	*HECTD1*	SNV	p.(Arg672Met)	32	0	120	25	93	
14	31619346	T	C	*HECTD1*	SNV	p.(Arg672Gly)	21	0	116	26	90	
15	23932268	G	A	*NDN*	SNV	p.(Pro33Ser)	8	3	23	11	32	
17	40489789	T	A	*STAT3*	SNV	p.(Met213Leu)	13	0	76	12	32	EMT
17	77758222	C	T	*CBX2*	SNV	p.(Pro327Leu)	2	0	25	17	63	
18	48591806	G	A	*SMAD4*	SNV	p.(Trp323*)	43	0	22	13	33	EMT
19	10600464	C	A	*KEAP1*	SNV	p.(Gly464Val)	27	0	75	11	35	
20	61528167	G	GA	*DIDO1*	Frameshift Insertion	p.(Phe590Leufs*99)	NA	0	85	30	92	
22	29977055	C	T	*NIPSNAP1*	SNV	p.(Cys6Tyr)	22	0	19	2	10	
**ALK14**												
1	16074055	G	C	*TMEM82*	SNV	p.(Gln320His)	16	16	88	54	141	
1	150795726	G	C	*ARNT*	SNV	p.(Phe446Leu)	32	0	76	13	57	EMT
1	172634924	T	A	*FASLG*	SNV	p.(Leu205Gln)	2	14	94	226	250	EMT
1	172634933	G	A	*FASLG*	SNV	p.(Ser208Asn)	10	13	87	225	250	EMT
1	172634968	C	G	*FASLG*	SNV	p.(Gln220Glu)	0	10	72	215	249	EMT
1	172634980	A	C	*FASLG*	SNV	p.(Met224Leu)	5	9	69	205	245	EMT
1	172634982	G	A	*FASLG*	SNV	p.(Met224Ile)	13	9	68	209	250	EMT
1	172634990	G	A	*FASLG*	SNV	p.(Gly227Glu)	11	9	71	211	249	EMT
1	172634996	T	G	*FASLG*	SNV	p.(Met229Arg)	3	7	73	200	240	EMT
1	172634998	A	T	*FASLG*	SNV	p.(Met230Leu)	7	6	72	200	242	EMT
1	172635002	G	A	*FASLG*	SNV	p.(Ser231Asn)	2	6	73	204	249	EMT
1	172635024	G	A	*FASLG*	SNV	p.(Met238Ile)	7	15	78	212	250	EMT
1	172635032	G	A	*FASLG*	SNV	p.(Arg241His)	6	16	85	209	249	EMT
1	180062254	G	A	*CEP350*	SNV	p.(Met2338Ile)	0	0	53	4	15	
1	180910336	G	A	*KIAA1614*	SNV	p.(Arg1025His)	24	0	41	14	54	

1	202724554	C	G	*KDM5B*	SNV	p.(Leu497Phe)	19	0	80	7	19	
2	64779826	G	T	*AFTPH*	SNV	p.(Leu406Phe)	13	0	95	2	10	
2	159077181	C	A	*CCDC148*	SNV	p.(Met441Ile)	9	0	25	2	10	
2	174055794	C	G	*MLTK*	SNV	p.(Phe157Leu)	21	0	109	2	10	EMT
2	174055799	AC	A	*MLTK*	Frameshift Deletion	p.(His160Ilefs*41)	NA	0	109	2	10	EMT
2	241938334	C	G	*SNED1*	SNV	p.(Ala27Gly)	16	0	12	3	14	
3	38061829	C	T	*PLCD1*	SNV	p.(Glu38Lys)	9	0	95	35	146	Metabolism
3	99514816	G	A	*COL8A1*	SNV	p.(Glu691Lys)	18	0	71	21	68	
6	34026857	G	C	*GRM4*	SNV	p.(Phe307Leu)	0	0	46	15	60	
6	43748488	G	A	*VEGFA*	SNV	p.(Gly328Arg)	22	0	18	15	39	EMT
6	72892467	G	C	*RIMS1*	SNV	p.(Glu431Asp)	11	0	39	16	61	
6	129571258	TCC	T	*LAMA2*	Frameshift Deletion	p.(Pro596Serfs*13)	NA	0	82	2	10	EMT
10	26575335	A	T	*GAD2*	SNV	p.(Tyr433Phe)	25	0	66	2	10	Metabolism
10	26575337	G	T	*GAD2*	SNV	p.(Asp434Tyr)	24	0	65	2	10	Metabolism
10	67829157	C	T	*CTNNA3*	SNV	p.(Glu690Lys)	33	0	186	5	24	EMT
12	27809577	TAGA	T	*PPFIBP1*	Inframe Deletion	p.(Glu275del)	NA	0	43	2	10	
12	75816737	GCA	G	*GLIPR1L2*	Frameshift Deletion	p.(Arg214Thrfs*2)	NA	0	27	2	10	
12	112888210	G	A	*PTPN11*	SNV	p.(Glu76Lys)	36	0	45	7	17	EMT
15	59934411	CAG	C	*GTF2A2*	Frameshift Deletion	p.(Leu76Glufs*2)	NA	0	69	2	10	
16	10631921	C	CTTC AGGG CCGG TCAG CTCA TTGA TCTC GGTA CAGT TT	*EMP2*	frameshift Insertion	p.(Leu61Phefs*73)	NA	1	26	28	64	
17	19246713	C	T	*B9D1*	SNV	p.(Met178Ile)	19	0	22	17	42	
17	56388384	G	A	*BZRAP1*	SNV	p.(Ser1091Leu)	9	4	30	12	43	
19	8922711	T	TAAG	*ZNF558*	Inframe Insertion	p.(Asn152_Asn15 2delinsThrTyr)	NA	0	48	2	10	

19	14184014	C	T	*MISP3*	SNV	p.(Pro107Ser)	12	0	49	17	56	
19	56538437	C	T	*NLRP5*	SNV	p.(Arg280Cys)	10	0	164	75	250	
20	57042661	G	T	*APCDD1L*	SNV	p.(Pro92His)	14	0	59	17	84	
X	57358218	G	T	*FAAH2*	SNV	p.(Gln200His)	7	4	25	7	13	
X	153069641	G	A	*PDZD4*	SNV	p.(Arg493Trp)	14	0	22	10	37	
**ALK16**												
1	85656064	T	A	*SYDE2*	SNV	p.(Asn373Tyr)	23	0	92	9	38	
1	104118133	C	A	*AMY2B*	SNV	p.(Arg358Ser)	6	0	24	2	10	Metabolism
2	31399464	G	A	*CAPN14*	SNV	p.(Gln673*)	38	2	29	19	83	
3	152132776	ATT	A	*MBNL1*	Frameshift Deletion	p.(Leu75Lysfs*11)	NA	0	190	2	10	
3	152132779	T	A	*MBNL1*	SNV	p.(Leu75*)	45	0	198	2	10	
5	58571190	CT	AGA	*PDE4D*	Frameshift Insertion	p.(Ser14Phefs*11)	NA	0	75	3	11	Metabolism
7	72984907	C	A	*TBL2*	SNV	p.(Arg425Leu)	22	2	47	18	69	
7	150498790	G	T	*TMEM176A*	SNV	p.(Ser51Ile)	14	1	28	16	57	
11	61508817	G	A	*DAGLA*	SNV	p.(Val723Ile)	13	1	11	8	21	
12	6840167	C	A	*COPS7A*	SNV	p.(Leu264Ile)	20	1	26	9	31	
14	24772980	G	A	*NOP9*	SNV	p.(Val443Met)	18	7	77	15	67	
14	81251682	TATGG	T	*CEP128*	Frameshift Deletion	p.(His589Aspfs*6)	NA	0	50	2	10	
19	40885573	A	G	*HIPK4*	SNV	p.(Leu591Pro)	14	2	10	6	15	

[1]: Annotation of mutations according to ALAMUT and mutalyzer https://mutalyzer.nl/; [2]: EMT, epithelial-mesenchymal transition.

Supplementary Table S3. Significantly enriched pathways harboring treatment-related variants in patients ALK6, ALK14 and ALK16.

Pathway name	Patient	Genes mutated	Enrichment score	Enrichment p-value
Proteoglycans in cancer	ALK6, ALK14	*ANK2, FASLG, HSPG2, PTPN11, STAT3, VEGFA*	8.0	0.00
Pathways in cancer	ALK6, ALK14	*ARNT, CTNNA3, FASLG, LAMA2, SMAD4, STAT3, VEGFA*	6.5	0.00
AGE-RAGE signaling pathway in diabetic complications	ALK6, ALK14	*PLCD1, SMAD4, STAT3, VEGFA*	6.4	0.00
Renal cell carcinoma	ALK14	*ARNT, PTPN11, VEGFA*	5.4	0.00
Hepatitis B	ALK6, ALK14	*FASLG, HSPG2, SMAD4 STAT3*	5.3	0.00
Pancreatic cancer	ALK6, ALK14	*SMAD4, STAT3, VEGFA*	5.0	0.01
HIF-1 signaling pathway	ALK6, ALK14	*ARNT, STAT3, VEGFA*	4.3	0.01
Type I diabetes mellitus	ALK14	*FASLG, GAD2*	4.2	0.01
FoxO signaling pathway	ALK6, ALK14	*FASLG, SMAD4, STAT3*	3.7	0.03
GABAergic synapse	ALK6, ALK14	*GAD2, SLC6A1*	3.4	0.03
Arrhythmogenic right ventricular cardiomyopathy	ALK14	*CTNNA3, LAMA2*	3.3	0.04
Taurine and hypotaurine metabolism	ALK14	*GAD2*	3.3	0.04
ECM-receptor interaction	ALK6, ALK14	*HSPG2, LAMA2*	3.3	0.04
Aldosterone synthesis and secretion	ALK6, ALK16	*DAGLA, SCARB1*	3.1	0.05
Adipocytokine signaling pathway	ALK6, ALK14	*PTPN11, STAT3*	3.1	0.05

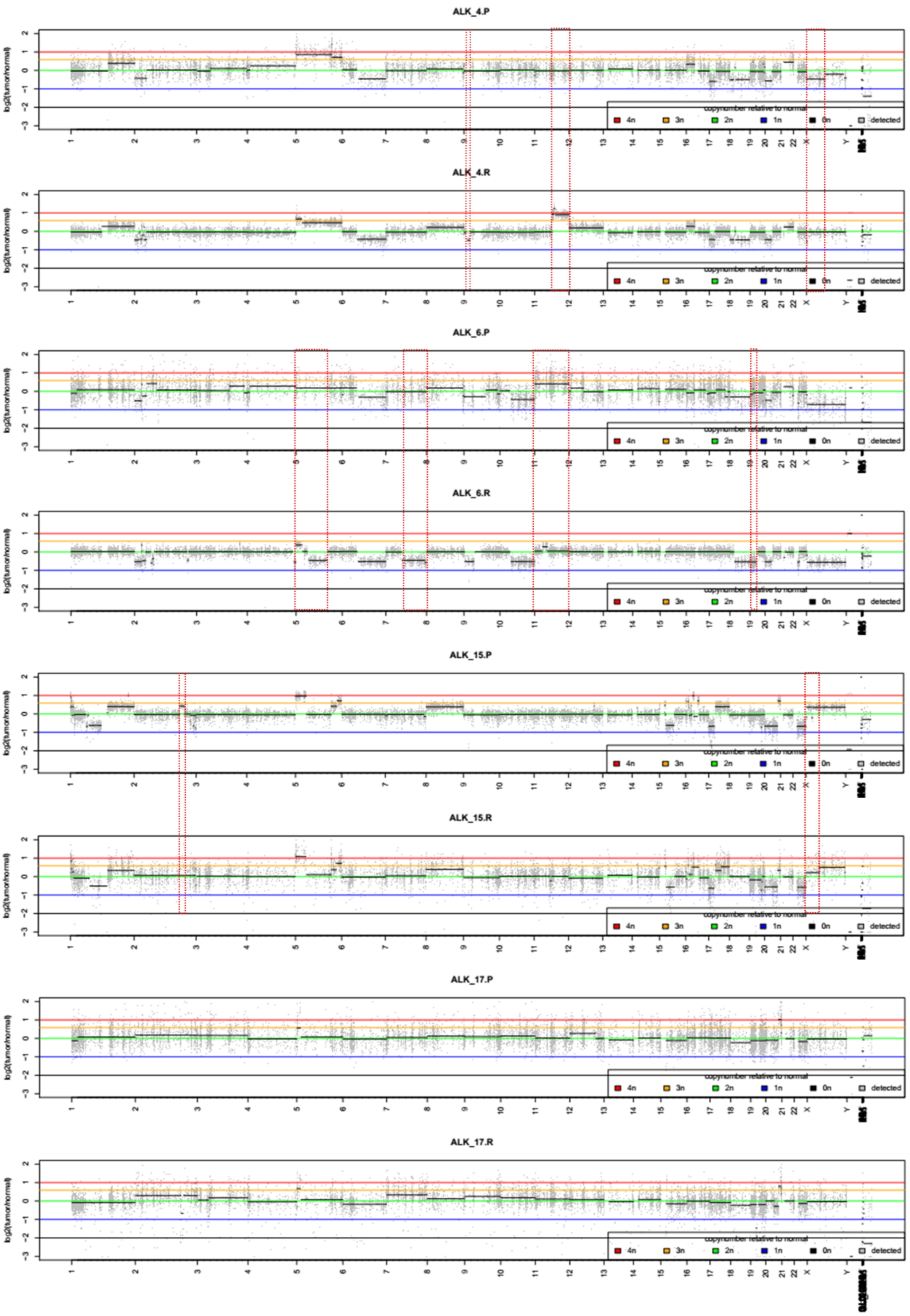

Supplementary Figure S1. Copy number variant plots across four patients with ALK rearranged advanced NSCLC with matched primary and resistant tumor samples. The chromosomal segments with conspicuous copy number differences between primary and resistant samples in each patient are marked by the red boxes.
